# Thermal Performance Curves Are Shaped by Prior Thermal Environment in Early Life

**DOI:** 10.3389/fphys.2021.738338

**Published:** 2021-10-20

**Authors:** Adriana P. Rebolledo, Carla M. Sgrò, Keyne Monro

**Affiliations:** School of Biological Sciences, Monash University, Melbourne, VIC, Australia

**Keywords:** climate change, carryover effects, complex life cycles, developmental plasticity, fertilization, embryogenesis, larval development, thermal sensitivity

## Abstract

Understanding links between thermal performance and environmental variation is necessary to predict organismal responses to climate change, and remains an ongoing challenge for ectotherms with complex life cycles. Distinct life stages can differ in thermal sensitivity, experience different environmental conditions as development unfolds, and, because stages are by nature interdependent, environmental effects can carry over from one stage to affect performance at others. Thermal performance may therefore respond to carryover effects of prior thermal environments, yet detailed insights into the nature, strength, and direction of those responses are still lacking. Here, in an aquatic ectotherm whose early planktonic stages (gametes, embryos, and larvae) govern adult abundances and dynamics, we explore the effects of prior thermal environments at fertilization and embryogenesis on thermal performance curves at the end of planktonic development. We factorially manipulate temperatures at fertilization and embryogenesis, then, for each combination of prior temperatures, measure thermal performance curves for survival of planktonic development (end of the larval stage) throughout the performance range. By combining generalized linear mixed modeling with parametric bootstrapping, we formally estimate and compare curve descriptors (thermal optima, limits, and breadth) among prior environments, and reveal carryover effects of temperature at embryogenesis, but not fertilization, on thermal optima at completion of development. Specifically, thermal optima shifted to track temperature during embryogenesis, while thermal limits and breadth remained unchanged. Our results argue that key aspects of thermal performance are shaped by prior thermal environment in early life, warranting further investigation of the possible mechanisms underpinning that response, and closer consideration of thermal carryover effects when predicting organismal responses to climate change.

## Introduction

For ectotherms, accounting for the vast majority of animals, population resilience to climate change rests on the capacity to maintain critical physiological functions that buffer performance, and ultimately fitness (survival and reproduction), against variation in environmental temperature (Deutsch et al., 2008; Sinclair et al., 2016). Changes in temperature need not be detrimental if they shift an organism’s performance closer to its thermal optimum, or shift the optimum itself (Angilletta et al., 2010; Sørensen et al., 2018). Within generations, such shifts can emerge due to phenotypic plasticity, with evidence suggesting that ectotherms can often remodel their physiology to compensate for chronic or recurring changes in temperature (Seebacher et al., 2015; Sgrò et al., 2016), or to directional selection screening differences in survival or reproduction at different temperatures (Donelson et al., 2018). These mechanisms may often be inseparable (indeed, effects of selection may often be attributed to plasticity; Donelson et al., 2018) and both can result in environmental effects carrying over from one life stage to affect fitness outcomes at others (Donelson et al., 2018; Moore and Martin, 2019). Consequently, there is considerable interest in how such carryover effects might impact population resilience to climate change (Dupont et al., 2013; Seebacher et al., 2015; Campbell et al., 2020). Understanding their nature, direction, and strength, however, remains an ongoing challenge due to the complex life cycles of many ectotherms, and may benefit from new insights into thermal performance curves at understudied life stages that limit resilience (Sinclair et al., 2016; Kingsolver and Buckley, 2020; Rebolledo et al., 2020).

Temperature does not affect the same organism equally at all life stages (Angilletta, 2009). For ectotherms with complex life cycles, distinct developmental stages separated by days or even less can differ in thermal sensitivity due to multiple factors (e.g., evolved differences in thermal optima, along with rapid changes in complexity, size, or duration of exposure to thermal challenges), and thermal challenges can vary in intensity from one stage to the next (Kingsolver et al., 2011; Freda et al., 2017; Ezeakacha and Yee, 2019; Rebolledo et al., 2020). Nevertheless, most studies to date measure thermal performance and sensitivity at single life stages (Byrne et al., 2020) and predominantly in adults (Truebano et al., 2018; Pandori and Sorte, 2019). This is problematic in light of emerging evidence that reproductive stages and embryos tend to be more thermally sensitive and may better predict the vulnerability of ectotherms to climate warming (Dahlke et al., 2020; Rebolledo et al., 2020; Collin et al., 2021; Van Heerwaarden and Sgrò, 2021). Moreover, thermal performance at these critical stages is often incompletely characterized due to well-known challenges in gathering sufficient data, so that information about ontogenetic shifts in thermal limits and thermal optima, in particular, currently remains too limited to identify any general patterns (Kingsolver and Buckley, 2020).

Life stages are by nature interdependent, and there is growing evidence that prior thermal environments can have lasting effects on performance later in life (Arambourou et al., 2017; Ezeakacha and Yee, 2019; Carter and Sheldon, 2020). Evidence also suggests that these carryover effects can be more lasting and pervasive the earlier that they are induced in ontogeny, and especially when induced at embryogenesis (Watkins and Vraspir, 2006; Jonsson and Jonsson, 2014; Noble et al., 2018). This outcome possibly relates to the particular thermal sensitivity of embryos (Sanger et al., 2018; Rebolledo et al., 2020; Collin et al., 2021), and ample scope for thermal perturbation of cell division, differentiation, and regulatory pathways during this window of development to profoundly alter future form, function, and performance (Van Der Have, 2002; Hamdoun and Epel, 2007; Begasse et al., 2015). In general, however, the adaptive significance of carryover effects – at least those attributable to plasticity – remains contentious. Prior exposure to a given temperature is often assumed to optimize future performance at the same temperature (the so-called beneficial acclimation hypothesis), but this assumption has been subject to much debate (Huey et al., 1999; Loeschcke and Hoffmann, 2002; Wilson and Franklin, 2002; Deere and Chown, 2006), and evidence remains equivocal (e.g., Sgrò et al., 2016; Sørensen et al., 2016; Brahim et al., 2019; Van Heerwaarden and Kellermann, 2020). It might be that carryover effects are more nuanced and alter other aspects of thermal performance, but again, few studies have explored effects of early thermal environments on performance curves (but see Seebacher and Grigaltchik, 2014) and, to our knowledge, effects induced at fertilization – the key life stage linking one generation to the next – have received little attention in this context (Walsh et al., 2019; Chirgwin et al., 2021).

Thermal performance curves explicitly relate changes in temperature to performance, whether measured in terms of physiological rates, growth or development rates, or fitness components such as survival, fecundity, or fertility (Sinclair et al., 2016; Kingsolver and Buckley, 2020). Curve shape can vary with the measure considered, with curves for rates tending to be skewed and curves for survival tending toward symmetry (Van Der Have, 2002; Kingsolver et al., 2011). Regardless, thermodynamic effects on physiology see performance rise with increasing temperature from its lower thermal limit (*CT*_min_) to a peak (*P*_max_) at the thermal optimum (*T*_opt_), before loss of metabolic efficiency or disruption of proteins and membranes at higher temperatures see it fall again to its upper thermal limit (*CT*_max_). Thermal breadth (*T*_br_, the range where performance is at least 50 or 80% of *P*_max_) is then derived from these curve descriptors. Performance curves are key tools for assessing and predicting the responses of ectotherms to ongoing climate change, since the impacts of higher temperatures hinge on where, on the curve, conditions lie at present. Ectotherms may thrive, for example, if presently living below their thermal optima, or risk extinction if already living at or near their upper thermal limits (Seebacher et al., 2015; Sinclair et al., 2016; Pinsky et al., 2019).

Importantly, thermal performance curves are unlikely to be fixed for any performance measure, and determining how curves may themselves shift in response to environmental cues is also vital for understanding population responses to climate change (Angilletta, 2009; Sinclair et al., 2016). Multiple hypotheses have sought to explain coordinated shifts in curve shape and position along the temperature axis based on tension between thermodynamic constraints and mechanisms of thermal adaptation (Huey and Kingsolver, 1989; Huey et al., 1999; Izem and Kingsolver, 2005; Deere and Chown, 2006; Angilletta et al., 2010). Those hypotheses variously predict, for example, positive associations between peak performance and thermal optimum (“hotter-is-better” or “cooler-is-better”) or between thermal optimum and thermal limits (“hotter-colder”), and negative associations between peak performance and thermal breadth (“generalist-specialist”). Other hypotheses (including those centering on the benefits of acclimation or plasticity above) address the complex and diverse ways in which prior thermal experience may modify curve shape and position. To date, however, most evidence comes from plants, whereas responses for animals remain understudied (Angilletta, 2009; but see Deere and Chown, 2006; Seebacher and Grigaltchik, 2014) and so idiosyncratic as to evade prediction and synthesis (Sinclair et al., 2016). Hence, there is still a need to better understand how prior thermal experience affects thermal performance, particularly in early life for which knowledge is still scarce.

Here, we estimate and compare how thermal environments at fertilization and embryogenesis shape thermal performance curves at completion of planktonic development in an aquatic ectotherm – the externally-fertilizing tubeworm, *Galeolaria caespitosa*. Like most aquatic ectotherms, *Galeolaria* has planktonic gametes, embryos, and larvae that are dispersed passively by currents, undergo the key processes of fertilization and development in direct contact with the external environment, and are major bottlenecks for population resilience to climate change (Byrne, 2011; Pinsky et al., 2019; Walsh et al., 2019; Dahlke et al., 2020). These stages therefore present unique scope to assess how prior thermal experience alters performance at early life stages that govern adult abundances and dynamics. Using a split-cohort experimental design to standardize genetic backgrounds across stages, we factorially manipulate temperatures at fertilization (18 and 22°C) and embryogenesis (18, 20, and 22°C), then, for each combination of prior temperatures, measure thermal performance curves for survival of planktonic development (end of the larval stage). By combining generalized linear mixed modelling with parametric bootstrapping, we formally estimate and compare curve descriptors (thermal optima, limits, and breadth) among prior environments, and reveal new insights into the effects of those environments on thermal performance in early life.

## Materials and Methods

### Study Species and Sampling

*Galeolaria caespitosa* (henceforth *Galeolaria*) is a calcareous tubeworm native to rocky shores of southeastern Australia, where it acts as an ecosystem engineer by forming dense colonies of tubes that provide habitat and reduce abiotic stress for associated communities (Wright and Gribben, 2017). Sessile adults breed year-round by releasing sperm and eggs into the sea for external fertilization (Chirgwin et al., 2020). Embryos develop into functionally-independent larvae ~24h later, then larvae develop for another ~2–3weeks until rapid changes in size, morphology, and behavior signal onset of metamorphosis (readiness to settle and recruit into sessile populations; Marsden and Anderson, 1981). These early life stages are bottlenecks for persistence under thermal stress (Byrne, 2011; Walsh et al., 2019), and exposure to stress at one stage can influence responses to the same level of stress later on (Chirgwin et al., 2021). However, the sensitivity of thermal performance curves to prior thermal environments in early life is unknown for organisms with complex life cycles like *Galeolaria*.

We sampled adult *Galeolaria* between March and July 2019 from a natural population at Brighton, Port Phillip Bay, Victoria, where water temperature ranges from 9°C in winter to 24°C in summer (Chirgwin et al., 2018). The region is a marine hotspot that has warmed at more than four times the global average rate in recent decades, and temperature is expected to increase by ~2–5°C by the century’s end (Hobday and Lough, 2011; Hobday and Pecl, 2014). Adults were transferred in insulated aquaria to seawater tanks at Monash University, and acclimatized for 14days at the mean annual temperature (17°C; Chirgwin et al., 2017) to reduce any effects of environmental differences among collection dates before obtaining gametes for experiments (gametogenesis is continuous and gametes can ripen in less than this time).

### Experimental Overview

To explore how prior thermal environment alters thermal performance in early life, we factorially manipulated temperatures at fertilization (18 and 22°C) and embryogenesis (18, 20, and 22°C), then estimated thermal performance curves for survival of planktonic development (end of the larval stage). Survival to this point in the life cycle measures the proportion of initial offspring that ultimately become ready to settle and recruit to the adult population, and recruitment of new individuals is directly linked to population viability (Byrne, 2011). Temperatures at fertilization and embryogenesis were selected to bracket projected warming of 2–4°C by mid-to-late century (Hobday and Lough, 2011) and include the thermal optimum previously estimated for each stage (~21°C for fertilization and ~19°C for embryogenesis; Rebolledo et al., 2020). Thermal performance curves were based on 10 temperatures spanning the full performance range (10–28°C) and including the thermal optimum previously estimated for survival of larval development (~19°C; Rebolledo et al., 2020).

Thermal environment was manipulated, and performance assayed, in replicate vials of filtered, pasteurized seawater (loosely capped to allow oxygen flow) suspended upright in water baths. Baths were maintained at designated temperatures (±0.1C) using controlled immersion heaters (Grant Optima TX150) for those ≥13°C and a refrigerated circulator (Julabo FP50) for 10°C. Four replicates were completed for each combination of temperatures with the exception of 27°C, for which two replicates were completed. Within each replicate, 30 individuals were evaluated for successful completion of development, giving an experiment-wide total of nearly 7,000 individuals. Replicates were generated in an incomplete block design with temperatures assigned haphazardly to blocks and unreplicated within them. Each block consisted of gametes, embryos, and larvae from the same cohort of parents used in one replicate per combination of temperatures at fertilization and embryogenesis, assayed at 2–5 temperatures at larval development (it was not logistically feasible to assay the full set of larval temperatures at once). Hence, all replicates per block were assayed concurrently using different subsets of material from the same parents, under identical conditions aside from the manipulation of temperature (see details below). There were 10 blocks in total.

### Gamete Collection and Manipulation of Temperature at Fertilization

Gametes were collected from 15 males and 15 females per block to minimize male-female compatibility effects at fertilization and development (Marshall and Evans, 2005; Chirgwin et al., 2017). To collect gametes, each mature adult was extracted from its tube and placed in a dish with ~1ml of fresh filtered seawater at 17°C to spawn. Gametes were collected immediately after spawning, checked for quality based on appearance of eggs and motility of sperm, then pooled by sex and used within the hour before viability declines (Rebolledo et al., 2020). Pooled eggs were diluted to ~250 cells ml^−1^ before use. Pooled sperm were kept concentrated at ~10^7^ cells ml^−1^ to minimize activity-related aging before use (Kupriyanova, 2006; Chirgwin et al., 2020). To initiate fertilizations, 45ml of pooled eggs and 5ml of pooled sperm were transferred separately to designated test temperatures (18 or 22°C), given 30min to adjust, then combined at test temperatures. After 30 min of gamete contact (which maximizes fertilization success; Rebolledo et al., 2020), samples were rinsed through 0.25μm mesh with seawater to remove excess sperm, then re-suspended in fresh seawater.

### Manipulation of Temperature at Embryogenesis

About 1–2h after fertilization (depending on temperature at fertilization), samples of two-cell embryos were transferred to designated test temperatures (18, 20, or 22°C) so that temperatures at this stage were fully crossed with temperatures at fertilization. We used two-cell embryos to ensure that all embryos were exposed to test temperatures at a similar point in development, and because this was the earliest point that they could be distinguished from unfertilized eggs under a stereomicroscope. Embryos were maintained in sufficient seawater to avoid oxygen-limitation (Chirgwin et al., 2018) until completing development into actively swimming, feeding larvae ~24h later.

### Assays of Thermal Performance at Completion of Planktonic Development

Thirty larvae were randomly allocated to each of 10–20 vials per designated test temperature (10, 13, 16, 18, 20, 22, 24, 26, 27, or 28°C, with fewer vials allocated to temperatures above 20°C), so that temperatures at this stage were fully crossed with temperatures at fertilization and embryogenesis. Larvae were maintained in sufficient seawater (10ml) to avoid oxygen-limitation (Chirgwin et al., 2018) and fed a mix of microalgae *ad libitum* (~1×10^4^ cells ml^−1^ every 2nd day). After the 1st week (larvae do not complete development in this time; Rebolledo et al., 2020), one vial was sampled destructively each day to monitor completion of development (normal onset of metamorphosis into the sessile form; Marsden and Anderson, 1981). Monitoring continued for up to 3weeks depending on temperature, and ended when a final vial was observed in which all larvae had either died or successfully completed development. Each of the ~7,000 individuals in those final vials was then scored as 1 (denoting survival at completion of planktonic development) or 0 (denoting mortality beforehand), capturing the proportion of offspring ready to recruit to the adult population. No data came from individuals observed during monitoring, which was done simply to reliably identify the end of development, irrespective of development time.

### Modeling Thermal Performance Curves

We fitted thermal performance curves to binary survival data (with 1 denoting survival or 0 denoting mortality) using a binomial mixed-effects regression model fitted with Laplace approximation in the *lme4* package (version 1.1-26; Bates et al., 2015) for *R* 4.0.5.^1^ Based on the shape of unconstrained smoothers fitted to raw data (Supplementary Figure S1), survival was modeled as a cubic function of temperature using orthogonal polynomials. Prior temperatures at fertilization and embryogenesis, and all possible interactions with linear, quadratic, and cubic trends relating survival to temperature, were modeled as additional fixed effects. Block and final vial sampled within blocks were modeled as random effects. Model diagnostics were checked using the *DHARMa* package (version 0.4.1; Hartig, 2021) and showed no violations of assumptions. The significance of fixed effects was tested using Wald *X*^2^ tests (Bolker et al., 2009) in the *car* package (version 3.0-10; Fox and Weisberg, 2019). For significant effects, estimates of linear, quadratic, and cubic trends in survival were extracted from the model, and contrasted between prior temperatures using Tukey-adjusted pairwise contrasts, in the *emmeans* package (version 1.6.0; Lenth et al., 2021).

### Estimates and CIs of Curve Descriptors

We extracted standard descriptors of thermal performance curves for temperatures at embryogenesis from the binomial mixed-effects regression model (curves did not differ among temperatures at fertilization, so descriptors were not extracted at this level). Thermal optimum (*T*_opt_) was calculated as the temperature of peak survival (*P*_max_). Thermal breadth (*T*_br_) was calculated as the temperature range at which survival was equal or above 50% of its peak (following Sinclair et al., 2016). Breadth is also commonly calculated at equal or above 80% of peak performance, but we chose 50% to capture more of the shapes of curves, and because results were qualitatively unchanged when 80% was used. Both calculations gave similar results to thermal tolerance (*CT*_max_-*CT*_min_), so only breadth is presented here. Critical thermal limits (*CT*_min_ and *CT*_max_) were calculated as the lower and upper temperatures at which survival was 5% of its peak. This approach differs to classical measures based on acute limits, but was done because binary data may approach 0% *via* an asymptote and limit the biological meaning of *CT*_min_ and *CT*_max_ at complete mortality (Kellermann et al., 2019). Again, results were qualitatively unchanged when limits were calculated at complete mortality.

Last, to compare curve descriptors extracted from the regression model among prior temperatures at embryogenesis, we used parametric bootstrapping, implemented in the *boot* package (version 1.3-27; Canty and Ripley, 2021), to estimate the mean and 95% CI of each descriptor based on 1,000 bootstrap replicates of the regression model. We considered descriptors to differ significantly among prior temperatures if their 95% CIs did not overlap. Because this may be a conservative measure of differences between temperatures, we also calculated means and 95% CIs for pairwise comparisons of descriptors between temperatures (in this case, descriptors are significantly different if the 95% CI for their comparison excludes 0). Inferring significance this way gave similar results to inferring significance from overlapping intervals.

## Results

### Modeling Thermal Performance Curves

**Figure 1:**
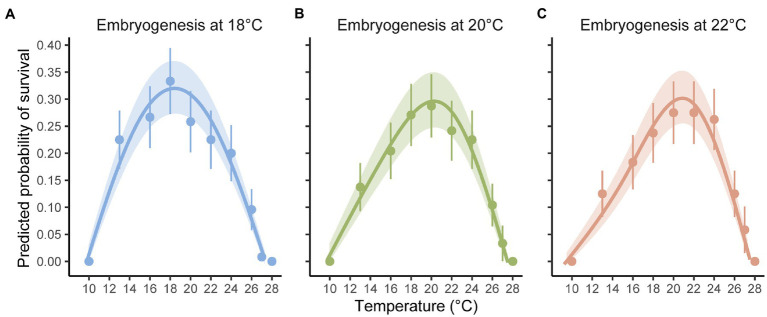
Thermal performance curves showing the predicted probabilities of successfully surviving planktonic development after embryogenesis at **(A)** 18, **(B)** 20, and **(C)** 22°C. Points are observed success (mean and 95% CI) per temperature. Curves are predicted from a binomial mixed-effects regression of success on temperature, with shaded areas indicating 95% CIs of curve predictions. Temperature at embryogenesis was manipulated factorially with temperature at fertilization, but no effects of fertilization history on thermal performance were detected (see Table 1; Supplementary Figure S1).

**Figure 2:**
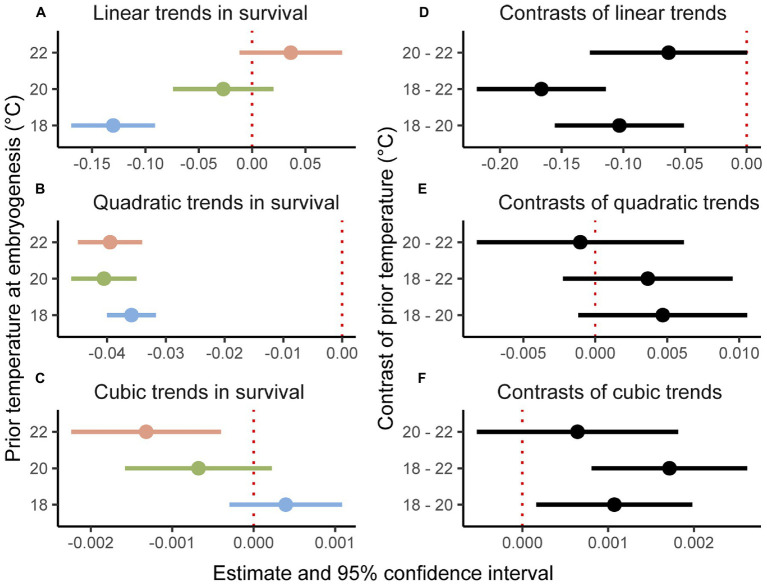
Linear, quadratic, and cubic trends **(A–C)** of thermal performance curves in Figure 1 contrasted **(D–F)** between prior temperatures at embryogenesis. Trends and contrasts differ significantly from 0 if their estimates have 95% credible intervals that exclude zero, marked by red dotted lines.

The binomial mixed-effects regression model gave a good overall fit to the data, and detected a significant interaction between temperature at embryogenesis and thermal performance in terms of survival of planktonic development (Figure 1; Table 1). Linear, quadratic, and cubic trends in survival extracted from the model (Figures 2A–C), and compared between temperatures using Tukey-adjusted pairwise contrasts (Figures 2D–F), attributed this interaction to shifts in linear and cubic trends in survival. Linear trends (estimating the average slopes of curves in Figure 1) shifted from negative after embryogenesis at 18°C to positive after embryogenesis at 22°C (Figure 2A), and differed significantly between 18°C and both of the other temperatures (the contrast between 20 and 22°C was marginally non-significant at *p*=0.12; Figure 2D). Cubic trends (estimating the degree to which slopes of curves in Figure 1 are steeper or shallower initially) shifted from positive after embryogenesis at 18°C to negative after embryogenesis at 22°C (Figure 2C), capturing differences in curvature to the left of peaks in Figure 1. Again, trends in survival differed significantly between 18°C and both other temperatures (Figure 2F). Quadratic trends (estimating the concavity of curves in Figure 1) were consistently negative (Figure 2B) and did not differ between temperatures (Figure 2E). Temperature at embryogenesis did not affect curve height (peak survival), indicated by its non-significant main effect in Table 1.

**Table 1:** Effects of prior temperatures at fertilization and embryogenesis on thermal performance in terms of survival at completion of planktonic development (modeled as a cubic function of temperature in a binomial mixed-effects regression model).

Fixed effects	*X* ^2^	d.f.	*p*
Temperature at fertilization	0.64	1	0.42
Temperature at embryogenesis	0.96	2	0.62
Temperature at fertilization×temperature at embryogenesis	1.30	2	0.53
Thermal performance at completion of planktonic development	460.35	3	**<0.001**
Temperature at fertilization×thermal performance	0.49	3	0.92
Temperature at embryogenesis×thermal performance	52.18	6	**<0.001**
Temperature at fertilization×temperature at embryogenesis×thermal performance	5.10	6	0.53

Significant effects are in bold. Linear, quadratic, and cubic trends in thermal performance are presented for prior temperatures at embryogenesis, and contrasted between temperatures, in Figure 2.

Temperature at fertilization did not affect survival of planktonic development or thermal performance at this stage in any way (all effects involving it were non-significant; Table 1).

### Estimates and CIs of Curve Descriptors

**Figure 3:**
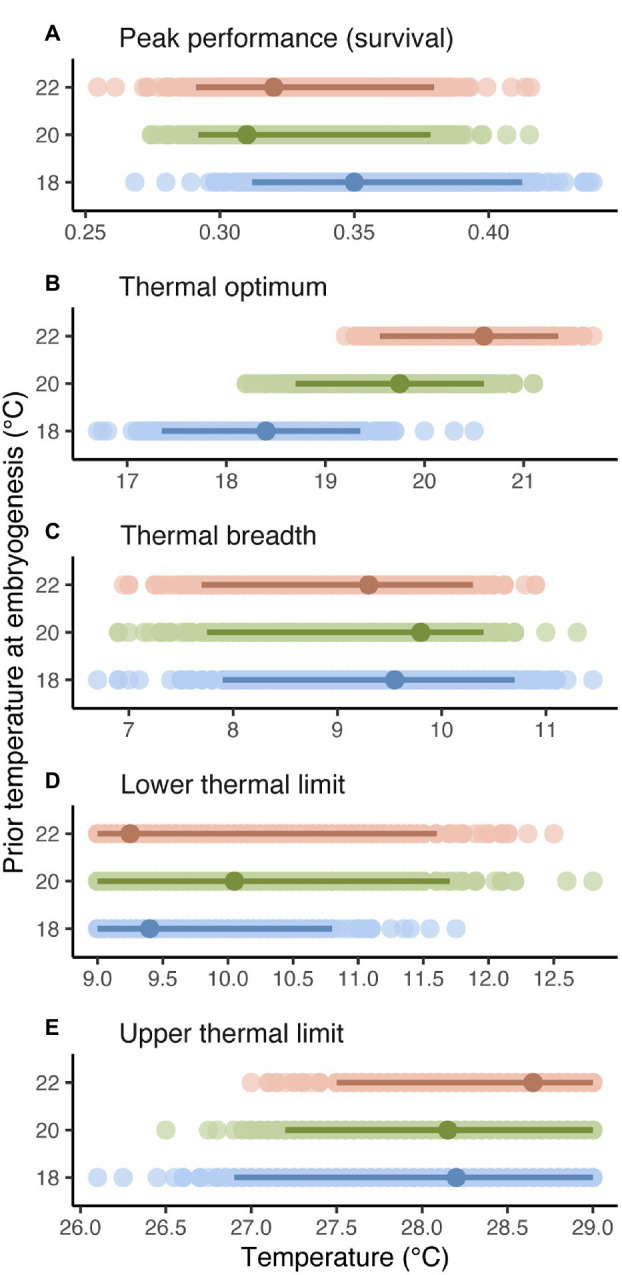
Peak performance (A), thermal optimum (B), thermal breadth (C), and thermal limits (D,E) for successful survival of planktonic development after embryogenesis at 18, 20, or 22°C (thermal performance was unaffected by fertilization at different temperatures before embryogenesis). Darker points and intervals are mean estimates and 95% CIs for curve descriptors, based on 1,000 bootstrap replicates (lighter points). See bootstrapping details in Materials and Methods.

As suggested by linear and cubic trends relating survival to temperature above, estimates and CIs for curve descriptors (Figure 3) showed that thermal optima for survival of planktonic development shifted to track prior temperature at embryogenesis (Figure 3B). Specifically, the estimated thermal optimum after embryogenesis at 18°C increased by 1.4°C after embryogenesis at 20°C and by another 0.9°C after embryogenesis at 22°C, and CIs for estimates did not overlap between 18 and 22°C. Note that these results may be somewhat conservative, given our cubic model tended to underestimate the thermal optimum at 22°C (Figure 1C; Supplementary Figure S1). Peak performance, thermal breadth, and thermal limits were unaffected by temperature at embryogenesis (Figures 3A,C–E).

## Discussion

Linking thermal performance to prior thermal experience is necessary to better understand and predict organismal responses to climate change. For ectotherms with complex life cycles this remains an ongoing challenge since life stages can differ in thermal sensitivity and experience different environmental conditions as development unfolds (Rebolledo et al., 2020). Seeing that life stages are by nature interconnected, environmental effects can carry over from one stage to affect performance at others (Arambourou et al., 2017; Lea et al., 2017; Ezeakacha and Yee, 2019). Thermal performance may therefore respond to carryover effects of prior thermal environments, yet detailed insights into the nature, strength, and direction of those responses are still lacking (Byrne et al., 2020). Here in *Galeolaria*, an aquatic ectotherm whose planktonic stages (gametes, embryos, and larvae) are considered most vulnerable to thermal stress (Pinsky et al., 2019; Walsh et al., 2019; Dahlke et al., 2020), we factorially manipulated temperatures at fertilization and embryogenesis, then, for each combination of prior temperatures, measured and compared thermal performance curves for survival at the end of planktonic development. Curves were unresponsive to temperature at fertilization, but temperature at embryogenesis caused shifts in larval thermal optima pointing to important carryover effects of thermal experience in this key window of development on survival.

Overall, the optimal temperature for completing 2–3weeks of planktonic development tracked the temperature experienced in 24h of embryogenesis beforehand, and did so without compromising peak performance (the proportion of larvae surviving development). To the extent that temperatures at embryogenesis and larval development match in nature, this carryover effect on thermal performance may increase individual fitness under modest levels of warming within the ~2–5°C range projected for the end the century (Hobday and Lough, 2011; Hobday and Pecl, 2014). Consequently, population viability may also be enhanced, since warming due to climate change is linked to changes in larval dispersal and recruitment that drive adult abundances and dynamics (Przeslawski et al., 2008). Enhanced population viability could further impact community structure, since *Galeolaria* is an ecosystem engineer that provides habitat for associated species (Wright and Gribben, 2017). Nevertheless, the extent to which prior thermal environment can buffer thermal performance in early life, and therefore have broader ecological impacts, seems to have its limitations, given that a 4°C increase in temperature at embryogenesis shifted the subsequent thermal optimum by only 2.2°C, and left thermal limits and breadth unchanged. Previous studies on terrestrial ectotherms have likewise reported limited scope for upper thermal limits of adults to increase in response to developmental temperature (Terblanche and Chown, 2006; Mitchell et al., 2011; Van Heerwaarden et al., 2016; Kellermann et al., 2017), although lower thermal limits tend to be more flexible (Araújo et al., 2013; Kingsolver and Buckley, 2020; Bennett et al., 2021). What exactly constrains thermal limits, and whether other descriptors of thermal performance are less constrained by comparison, remains actively debated (Schulte, 2015). Our results for *Galeolaria* show that the thermal optimum for planktonic development, at least, can respond to temperature at embryogenesis independently of other descriptors of thermal performance, and despite apparent constraints on upper limits.

Embryogenesis is the most formative life stage (Noble et al., 2018) and it is emerging as a critical threshold of thermal sensitivity in ectotherms whose embryos have no alternative but to develop in direct contact with the external environment (Van Der Have, 2002; Hamdoun and Epel, 2007; Begasse et al., 2015; Dahlke et al., 2020; Rebolledo et al., 2020). Consequently, carryover effects of temperature at this stage can be profound and persist across the life cycle (Watkins and Vraspir, 2006; Noble et al., 2018). The cellular mechanisms underlying such effects are poorly understood, but much attention has focused on inducible stress-response proteins that are differentially expressed in early life (Sørensen et al., 2003; Hammond and Hofmann, 2010; Burton and Metcalfe, 2014; Lockwood et al., 2017). Parents can load such proteins into waterborne gametes before release (Hamdoun and Epel, 2007; Hammond and Hofmann, 2010), potentially buffering gametes against direct thermal stress (Rebolledo et al., 2020) and explaining the lack of carryover effects of temperature at fertilization on thermal performance here. Embryos seem to downregulate these proteins when cell division is most active and overexpression is detrimental (Sørensen et al., 2003; Hamdoun and Epel, 2007), but shift to upregulation in response to thermal stress once cells start to differentiate and robust developmental pathways become vital (Leemans et al., 2000; Brown et al., 2004; Hamdoun and Epel, 2007; Hammond and Hofmann, 2010; Lockwood et al., 2017). Few studies, to our knowledge, have explicitly linked the induction of stress-response proteins at one life stage to carryover effects on thermal performance at others (Boon-Niermeyer et al., 1988; Hammond and Hofmann, 2010), but this is one mechanism by which prior exposure to stress may enhance performance under future stress (Sørensen et al., 2003) and a plausible reason why higher temperatures at embryogenesis might prime larvae to have higher thermal optima here.

Whatever the underlying mechanism, carryover effects in early life are widely attributed to developmental plasticity – that is, changes in gene expression triggered by environmental cues at development and often interpreted as epigenetic in origin (Beldade et al., 2011; Beaman et al., 2016; Bonamour et al., 2019). Developmental plasticity can be adaptive if it enhances later fitness in the environment that triggered it, but can also be nonadaptive or maladaptive if, for example, cues are unpredictable, or organisms cannot sense and respond to cues fast enough for effective environmental matching (Beaman et al., 2016; Bonamour et al., 2019). Despite ongoing interest in thermal developmental plasticity as a means for ectotherms to cope with climate change (Sgrò et al., 2016; Donelson et al., 2018; Noble et al., 2018; Carter and Sheldon, 2020; Rodrigues and Beldade, 2020), evidence for adaptive plasticity in thermal performance triggered by temperature at embryogenesis rests primarily on physiological measures of performance (e.g., Scott and Johnston, 2012; Seebacher and Grigaltchik, 2014; Refsnider et al., 2019), while measures with closer links to fitness (survival and reproduction) are less studied. Here in *Galeolaria*, enhanced survival at temperatures experienced at embryogenesis appears to be broadly consistent with adaptive developmental plasticity, but also raises the prospect of viability selection as an alternative or added explanation.

In ectotherms with complex life cycles, episodes of selection in early life can potentially combine to shape genetic composition at later stages, allowing carryover effects to have fitness outcomes not purely driven by plasticity (Moore and Martin, 2019). This may be especially likely for external fertilizers like *Galeolaria*, which produce numerous propagules with high intrinsic mortality at successive planktonic stages, in addition to direct exposure to environmental stressors (Foo and Byrne, 2016; Chirgwin et al., 2020; Crean and Immler, 2021). It is therefore possible that our manipulations of temperature at fertilization and embryogenesis screened each stage by differential survival at different temperatures, and that subsequent increases in thermal optima for survival reflect shifts in allele frequencies, not just expression, driven by directional selection. *Galeolaria* may have limited scope to respond evolutionarily, however, based on recent evidence that genetic variation for survival to independence (capacity to swim and feed, overlapping our performance measure here) is negligible after fertilization and embryogenesis at 24°C (Chirgwin et al., 2021). Disentangling selection and developmental plasticity as candidate drivers of carryover effects is notoriously hard to do experimentally, and may ultimately require genomic approaches (Donelson et al., 2018; Fox et al., 2019). In the meantime, we cannot be certain whether plasticity or selection, or both drivers in combination, underpin the carryover effects on thermal optima detected here.

Overall, our work reveals carryover effects of temperature at embryogenesis (but not fertilization) on thermal performance in early life that may buffer vulnerable planktonic stages of aquatic ectotherms against climate change, and offers new insights into the responses of thermal performance curves to thermal history. In particular, curve descriptors did not respond to temperature in the coordinated manner predicted by hypotheses based on thermodynamic constraints – that is, higher thermal optimum did not coincide with higher peak performance (as suggested by the “hotter-is-better” hypothesis; Huey and Kingsolver, 1989; Angilletta et al., 2010; Sørensen et al., 2018), or with horizontal shifts in thermal limits (as suggested by the “hotter-colder” hypothesis; Izem and Kingsolver, 2005; Angilletta, 2009). Of similar “rules” (or variants on them) invoked to explain how curves respond to prior thermal experience (Huey et al., 1999; Deere and Chown, 2006), our results seem most consistent with an interpretation of the beneficial acclimation hypothesis that assumes no covariation between thermal optimum and peak performance. This is termed temperature compensation (partial or complete maintenance of physiological rates in the face of changing temperature) and may be the combined outcome of thermal adaptation and thermodynamic constraints (Clarke, 2003). Such an interpretation is of course speculative at this point. Our results do, however, add to mounting evidence pointing to embryogenesis as the most critical of early life stages in aquatic ectotherms, not only for the emergence of thermal sensitivity (Dahlke et al., 2020; Rebolledo et al., 2020; Collin et al., 2021), but also of thermal carryover effects. Although, our results suggest that fertilization matters less in this regard, the possibility remains that the environment at gametogenesis is more influential than the environment at fertilization, emphasising the need to better understand transgenerational effects on thermal performance. Further research is therefore needed to elucidate how parental and developmental environments interact to shape thermal performance in organisms with complex life cycles, and thereby gain a clearer picture of organismal responses and vulnerability to current and future climatic conditions.

## Data Availability Statement

The raw data supporting the conclusions of this article will be made available by the authors, without undue reservation.

## Author Contributions

AR, CS, and KM conceived and designed the study. AR collected the data. AR and KM performed analyses and drafted the manuscript. KM created the graphics. All authors contributed to the article and approved the submitted version.

## Funding

This research was supported by a Holsworth Wildlife Research Endowment awarded to AR, and by grants awarded under the Australian Research Council’s Discovery Scheme to KM and CS.

## Conflict of Interest

The authors declare that the research was conducted in the absence of any commercial or financial relationships that could be construed as a potential conflict of interest.

## Publisher’s Note

All claims expressed in this article are solely those of the authors and do not necessarily represent those of their affiliated organizations, or those of the publisher, the editors and the reviewers. Any product that may be evaluated in this article, or claim that may be made by its manufacturer, is not guaranteed or endorsed by the publisher.

## Footnotes

^1^ https://www.r-project.org/

## Supplementary Material

Supplementary Figure S1 Proportions of individuals that successfully survived planktonic development after fertilization at 18 or 22°C and embryogenesis at 18, 20, or 22°C. Points are mean success (with bars indicating 95% CIs) and curves are locally-weighted smoothers (with shaded areas indicating 95% CIs) that assume no particular shape.
