# Supplementary figures and images for: Thermal Performance Curves Are Shaped by Prior Thermal Environment in Early Life
Source: Front Physiol. 2021 Oct 20;12:738338. doi: 10.3389/fphys.2021.738338 (PMC8564010; doi:10.3389/fphys.2021.738338)

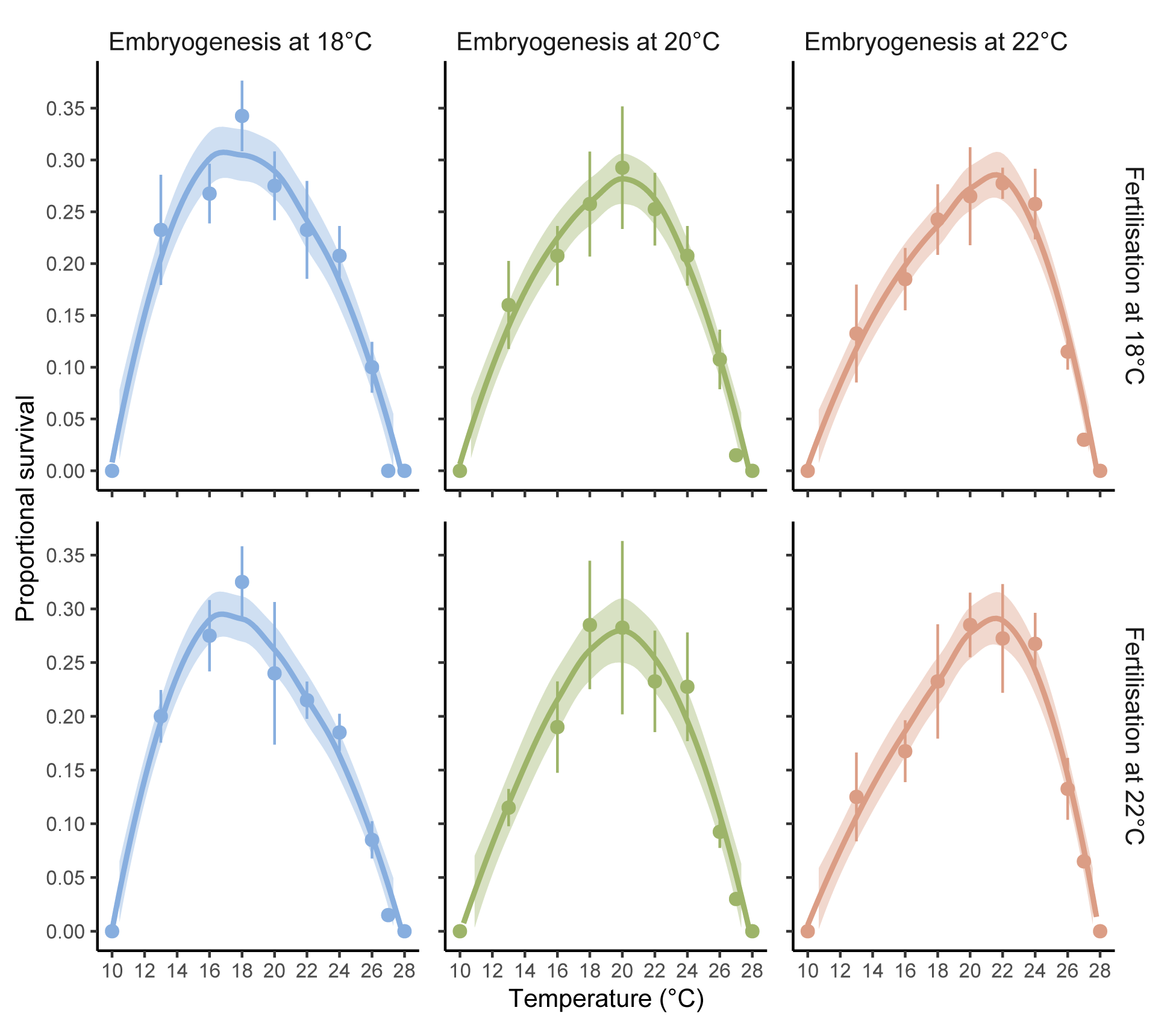

Supplement: Supplementary Figure S1 — Proportions of individuals that successfully survived planktonic development after fertilization at 18 or 22°C and embryogenesis at 18, 20, or 22°C. Points are mean success (with bars indicating 95% CIs) and curves are locally-weighted smoothers (with shaded areas indicating 95% CIs) that assume no particular shape. [file Image_1.TIF]
